# Sporotrichosis in Rio de Janeiro, Brazil: *Sporothrix brasiliensis* Is Associated with Atypical Clinical Presentations

**DOI:** 10.1371/journal.pntd.0003094

**Published:** 2014-09-18

**Authors:** Rodrigo Almeida-Paes, Manoel Marques Evangelista de Oliveira, Dayvison Francis Saraiva Freitas, Antônio Carlos Francesconi do Valle, Rosely Maria Zancopé-Oliveira, Maria Clara Gutierrez-Galhardo

**Affiliations:** 1 Laboratório de Micologia, Instituto de Pesquisa Clínica Evandro Chagas, Fundação Oswaldo Cruz, Rio de Janeiro, Rio de Janeiro, Brazil; 2 Laboratório de Dermatologia Infecciosa, Instituto de Pesquisa Clínica Evandro Chagas, Fundação Oswaldo Cruz, Rio de Janeiro, Rio de Janeiro, Brazil; University of California San Diego School of Medicine, United States of America

## Abstract

**Background:**

There have been several recent changes in the taxonomy of *Sporothrix schenckii* as well as new observations regarding the clinical aspects of sporotrichosis. In this study, we determined the identification of the *Sporothrix* species associated with both classic and unusual clinical aspects of sporotrichosis observed in the endemic area of sporotrichosis in Rio de Janeiro, Brazil.

**Methodology/Principal Findings:**

To verify whether *S. brasiliensis* is associated with clinical manifestations of sporotrichosis, a cross-sectional study was performed in which *Sporothrix* isolates from 50 patients with different clinical manifestations were analyzed and their isolates were studied by phenotypic and genotypic methods. Data from these patients revealed a distinct clinical picture and therapeutic response in infections caused by *Sporothrix brasiliensis* (n = 45) compared to patients with *S. schenckii* sensu stricto (n = 5). *S. brasiliensis* was associated with disseminated cutaneous infection without underlying disease, hypersensitivity reactions, and mucosal infection, whereas patients with *S. schenckii* presented with less severe and more often localized disease, similar to the majority of previously described sporotrichosis cases. Interestingly, *S. brasiliensis*-infected patients overall required shorter durations of itraconazole (median 16 weeks) compared to the individuals with *S. schenckii* (median 24 weeks).

**Conclusions/Significance:**

These findings suggest that *Sporothrix* species are linked to different clinical manifestations of sporotrichosis and that *S. brasiliensis* is effectively treated with oral itraconazole.

## Author Summary

*Sporothrix brasiliensis* is a dimorphic fungus that is responsible for an ongoing epidemic of cat-transmitted sporotrichosis in Rio de Janeiro, Brazil. More than 4,100 human cases have been diagnosed in only one health institution since 1998. Most patients are children or housewives with frequent contact with domestic and/or stray cats. The patients usually live under poverty conditions in suburban regions of the metropolitan area with poor access to health care and unsanitary living conditions. For instance, most patients report that they need to have cats in their houses as a control against invasion by rodents. It is important to study the clinical aspects of *S. brasiliensis* infection in order to improve patient management, including optimizing therapeutic and prophylactic approaches. We have found that *S. brasiliensis* is responsible for some unusual clinical manifestations of sporotrichosis, such as disseminated infection in immunocompetent patients and hypersensitivity reactions. Also, treatment with itraconazole appears to be extremely effective in most cases of infection by *S. brasiliensis*. Our study will contribute for the management of the infection caused by *S. brasiliensis*, bringing benefits to the patients with sporotrichosis.

## Introduction

Sporotrichosis is a subcutaneous mycosis with a worldwide distribution that is currently notable for areas of especially high endemicity in Latin America [1]–[3]. Some authors classify sporotrichosis as an implantation mycosis, because this infection may also involves other sites beyond the skin and the subcutaneous tissues, such as lymphatic vessels, muscles, fascia, cartilage, and bones [3]. Historically, sporotrichosis has been attributed to a single species, *Sporothrix schenckii* [1]. However, phenotypic and genotypic analyses by Marimon and coworkers [4] have led to the identification of four new species in the *Sporothrix* complex: (i) *S. globosa*, a globally distributed fungus [5]–[7]; (ii) *S. brasiliensis*, the species related to the zoonotic epidemic of sporotrichosis in Rio de Janeiro, Brazil [8]; (iii) *S. mexicana*, initially limited to Mexico [4], but with recent cases reported in other regions [9], [10]; and (iv) *S. luriei*, formerly *S. schenckii* var. *luriei* [11].

Classical infection is associated with traumatic subcutaneous inoculation of soil, plants, or organic matter contaminated with fungus, with rare cases of transmission occurring from infected animals [1]. However, in Rio de Janeiro state, Brazil, sporotrichosis is currently largely occurring via transmission from infected cats to humans [12]. Recently, our group performed a georeferencing survey of sporotrichosis cases that revealed a transmission belt along the border between Rio de Janeiro city and adjacent counties in the Greater Metropolitan Area [13]. Genotypic analyses have shown that isolates from the Rio de Janeiro epidemic have a high genetic similarity, which is suggestive of a common niche [14], [15].

Although some studies have described several clinical aspects of this epidemic [12], [16], [17], taxonomic analyses have not been correlated with disease presentations. Therefore, the main purpose of this study was to investigate a possible association between manifestations of sporotrichosis in Rio de Janeiro and the different genomic species of *S. schenckii* sensu lato.

## Materials and Methods

### Ethics Statement

This study was approved by the Research Ethics Committee of Fundação Oswaldo Cruz (FIOCRUZ), under the number CAAE-0024.0.009.000-10. All patient samples and data were analyzed anonymously after receiving a random number during database construction.

### Patients

A cross-sectional study was performed in 50 patients with different clinical forms of sporotrichosis. They were selected from a database of 246 patients [8] who had *Sporothrix* strains isolated from clinical specimens and stored at the Pathogenic Fungal Collection of the Laboratório de Micologia at IPEC, and which were part of a cohort of 1,563 patients with sporotrichosis treated from 1999 to 2008 at the Instituto de Pesquisa Clínica Evandro Chagas (IPEC). Patients were submitted to a protocol that included clinical evaluation, mycological examination of clinical specimens and blood tests (blood count, biochemistry and liver function). In the absence of disseminated disease, oral itraconazole 100 mg/day was prescribed. Higher intraconazole doses were used if the lesions worsened or remained unchanged after eight weeks. The duration of treatment was determined by clinical cure (lesion healing defined as epithelization and absence of crusts, infiltrates, or erythema). Clinical cure of extracutaneous sites was defined as the disappearance of preexisting lesions in cases of conjunctival, nasal, or oral mucosa involvement. Patients with disseminated sporotrichosis received amphotericin B at a total dose of 1–2.5 g. Follow-up was 4–12 weeks after clinical cure. The data were collected by review of medical charts and were recorded on a standardized case report form, containing demographic, epidemiologic, clinical and follow-up information. Since the Rio de Janeiro sporotrichosis outbreak is massive, we had to establish some criteria to select patients related to common and unusual manifestations of sporotrichosis. Inclusion criteria for selection in this study were: patients who lived in Rio de Janeiro city or in other cities from Rio de Janeiro state in Brazil, patients with common (fixed cutaneous and lymphocutaneous) and unusual (disseminated cutaneous, extracutaneous, and disseminated) clinical forms of sporotrichosis [1], patients with and without hypersensitivity manifestations (eythema nodosum or erythema multiforme), patients co-infected with HIV, and patients treated with itraconazole as well as patients with spontaneous regression of lesions. However, for the less common variables (e.g., patients outside the endemic area), all available cases were included.

### Strains

The fungal isolates were cultured from different body sites, such as skin, eyes, nose, or cerebrospinal fluid. Each isolate was previously identified by classical microbiological phenotypic techniques as *S. schenckii* sensu lato. Additionally, control strains CBS 120339 (*S. brasiliensis*) [4], ATCC 16345 (*S. schenckii*), IPEC 27135 (*S. globosa*) [7], and MUM 11.02 (*S. mexicana*) [9] were included in identification tests.

### Phenotypic Characterization

Filamentous fungal colonies for each isolate were grown on Sabouraud Dextrose Agar and slide cultures were mounted with Lactophenol Cotton Blue (Fluka Analyted, France) for *Sporothrix* identification [4]. Dimorphism was demonstrated by conversion to the yeast-like form on Brain Heart Infusion Agar slants for 7 days at 37°C. Furthermore, colonies were sub-cultured on Potato Dextrose Agar plates and Corn Meal Agar slants, and incubated at 30 and 37°C in the dark to study fungal growth and sporulation respectively [4], [8]. Carbohydrate assimilation tests were performed using freshly prepared yeast nitrogen base (YNB) medium supplemented with sucrose or raffinose, using YNB supplemented with glucose as positive control and YNB without carbohydrates as a negative control. Experiments were performed at least three times on different days and, in the case of discordant results, repeated two additional times. All culture media were from Difco (Becton, Dickinson and Company/Sparks MD, USA). Results were interpreted according to the identification key detailed by Marimon and coworkers [11].

### Molecular Identification

Genomic DNA was extracted and purified from *Sporothrix* spp mycelial phase by chloroform/isoamyl alcohol method as described [7]. The gene encoding for the nuclear calmodulin was used for molecular differentiation of the isolates because this locus has a high number of parsimony informative sites, allowing *Sporothrix* differentiation in several genotypes [18]. For partial sequencing of the nuclear calmodulin (CAL) gene, we used the primers CL1 (5′-GA(GA)T(AT)CAAGGAGGCCTTCTC-3′), and CL2A (5′-TTTTTGCATCATGAGTTGGAC-3′) under previously described conditions [7]. Automated sequencing was done using the Sequencing Platform at PDTIS/FIOCRUZ, Brazil [19]. Sequences from both DNA strands were generated and edited with the Sequencher ver. 4.6 software package (Genes Codes Corporation, USA), followed by alignment with Mega version 4.0.2 software. Our sequences were compared by BLAST (Basic Local Alignment Search Tool) with sequences available from NCBI GenBank (*Sporothrix* AM 398382.1/AM 398393.1/AM 117444.1/AM 116899.1/AM 116908.1). All phylogenetic analyses were performed as previously described [7], [8].

### Nucleotide Sequence Accession Numbers

All sequences from isolates included in genotypic analysis were deposited in the GenBank database under accession numbers GU456632, HQ426928 to HQ426962, and KC463890 to KC463903.

### Statistics

Data were processed and analyzed using the SPSS 17.0 software. Frequencies and median values were calculated for each group of this study.

## Results

### Patients

Of the 50 patients, 16 were male and 34 female, with ages ranging from 9 to 83 years (median = 47). Lesions were located at upper limbs (n = 31, 62%), lower limbs (n = 6, 12%), face (n = 1, 2%), trunk (n = 1, 2%), and more than one segment (n = 11, 22%). Fifteen patients (30%) presented with a fixed cutaneous form, 24 (48%) lymphocutaneous form, 6 (12%) disseminated cutaneous form, and 5 (10%) disseminated (involving internal tissues) sporotrichosis. Additionally, six of these patients also presented with erythema nodosum and four with erythema multiforme. Table 1 summarizes the clinical and mycological information for each patient.

**Table 1:** Clinical, epidemiological, and mycological aspects of 50 sporotrichosis cases.

						Genotypic characterization		
Strain	Cat*	Clinical form	Erythema	Treatment (weeks)	Phenotypic identification	Final identification	Genbank n°	References
16490	Yes	Lymphocutaneous		13	*S. brasiliensis*	*S. brasiliensis*	AM116899	4
16919	Yes	Lymphocutaneous		16	*S. brasiliensis*	*S. brasiliensis*	HQ426930	8
17307	Yes	Disseminated Cutaneous		20	*S. schenckii*	*S. brasiliensis*	KC463892	This study
17331	Yes	Disseminated Cutaneous		36	*S. brasiliensis*	*S. brasiliensis*	HQ426929	This study
17521	Yes	Lymphocutaneous		36	*S. brasiliensis*	*S. schenckii*	KC463901	This study
17585	Yes	Fixed		24	*S. schenckii*	*S. schenckii*	KC463902	This study
17786	Yes	Lymphocutaneous		36	*Sporothrix spp.*	*S. brasiliensis*	HQ426931	This study
17878	Yes	Fixed	EN^a^	12	*Sporothrix spp.*	*S. brasiliensis*	HQ426932	8
24372	No	Disseminated		44 (AIDS)	*S. schenckii*	*S. schenckii*	KC463903	This study
25011	Yes	Fixed	EM^b^	16	*S. brasiliensis*	*S. brasiliensis*	HQ426935	8
25303	No	Disseminated		260 (AIDS)	*S. schenckii*	*S. brasiliensis*	KC463891	This study
25374	Yes	Lymphocutaneous	EN	Lost	*S. brasiliensis*	*S. brasiliensis*	KC463894	This study
25457	Yes	Lymphocutaneous		Lost	*S. brasiliensis*	*S. brasiliensis*	KC463890	This study
25521	Yes	Disseminated		20	*Sporothrix spp.*	*S. brasiliensis*	HQ426936	8
25758	Yes	Lymphocutaneous		16	*S. brasiliensis*	*S. brasiliensis*	KC463895	This study
26611	Yes	Fixed	EM	16 (HIV)	*Sporothrix spp.*	*S. brasiliensis*	HQ426937	8
26938	Yes	Fixed		48	*Sporothrix spp.*	*S. brasiliensis*	HQ426938	8
26945	No	Lymphocutaneous		14	*Sporothrix spp.*	*S. brasiliensis*	HQ426939	8
26961	No	Fixed		24	*S. schenckii*	*S. schenckii*	JN995605	34
27022	Yes	Disseminated Cutaneous		8	*S. brasiliensis*	*S. brasiliensis*	HQ426940	8
27052	No	Fixed	EN	12	*Sporothrix spp.*	*S. brasiliensis*	HQ426941	8
27087	Yes	Lymphocutaneous		64	*S. brasiliensis*	*S. brasiliensis*	HQ426942	8
27100	Yes	Fixed		36	*S. schenckii*	*S. brasiliensis*	JN995609	34
27130	Yes	Lymphocutaneous		16	*Sporothrix spp.*	*S. brasiliensis*	HQ426943	8
27133	Yes	Fixed		Lost	*S. schenckii*	*S. brasiliensis*	JN995608	34
27177	Yes	Lymphocutaneous		6	*Sporothrix spp.*	*S. brasiliensis*	HQ426944	8
27209	Yes	Lymphocutaneous		12	*Sporothrix spp.*	*S. brasiliensis*	HQ426946	8
27288	Yes	Lymphocutaneous		104	*Sporothrix spp.*	*S. brasiliensis*	HQ426945	8
27372	Yes	Fixed	EM	12	*Sporothrix spp.*	*S. brasiliensis*	HQ426947	8
27375	Yes	Fixed	EN	SR^c^	*S. schenckii*	*S. brasiliensis*	KC463898	This study
27387	Yes	Lymphocutaneous		12	*Sporothrix spp.*	*S. brasiliensis*	HQ426948	8
27417	No	Lymphocutaneous		16	*Sporothrix spp.*	*S. brasiliensis*	HQ426949	8
27445	Yes	Disseminated Cutaneous		SR	*S. brasiliensis*	*S. brasiliensis*	HQ426950	8
27454	Yes	Lymphocutaneous		22	*S. brasiliensis*	*S. brasiliensis*	KC463896	This study
27558	Yes	Fixed		12	*S. schenckii*	*S. brasiliensis*	KC463899	This study
27722	No	Fixed		SR	*S. mexicana*	*S. schenckii*	HQ426961	8
27930	No	Lymphocutaneous		10	*Sporothrix spp.*	*S. brasiliensis*	HQ426951	8
28329	Yes	Lymphocutaneous		16	*S. schenckii*	*S. brasiliensis*	JN995610	34
28403	Yes	Lymphocutaneous		10	*Sporothrix spp.*	*S. brasiliensis*	KC463900	This study
28487	Yes	Disseminated Cutaneous	EN	16	*S. brasiliensis*	*S. brasiliensis*	HQ426928	8
28604	Yes	Lymphocutaneous		10	*S. brasiliensis*	*S. brasiliensis*	HQ426953	8
28665	Yes	Fixed		SR	*S. brasiliensis*	*S. brasiliensis*	JN995606	34
28701	Yes	Disseminated Cutaneous		20	*Sporothrix spp.*	*S. brasiliensis*	HQ426954	8
28772	Yes	Lymphocutaneous		12	*Sporothrix spp.*	*S. brasiliensis*	HQ426955	8
28790	Yes	Lymphocutaneous		4	*S. brasiliensis*	*S. brasiliensis*	HQ426956	8
28988	Yes	Lymphocutaneous	EM	12	*S. schenckii*	*S. brasiliensis*	KC463897	This study
30650	Yes	Disseminated	EN	16	*S. brasiliensis*	*S. brasiliensis*	KC463893	This study
33605	Yes	Disseminated		34 (AIDS)	*Sporothrix spp.*	*S. brasiliensis*	HQ426957	8
33722	Yes	Lymphocutaneous		12	*Sporothrix spp.*	*S. brasiliensis*	HQ426958	8
34007	Yes	Fixed		Lost	*Sporothrix spp.*	*S. brasiliensis*	HQ426959	8

*indicates exposure (Yes) or no exposure (No) to cats.

a EN: erythema nodosum.

b EM : erythema multiforme.

c SR: spontaneous regression of lesions.

### Mycological and Phenotypic Identification

Of the 50 strains, 45 (90%) were classified by molecular methods as *S. brasiliensis* and 5 (10%) as *S. schenckii*. In 21 (42%) isolates, results from phenotypic tests were inconclusive, precluding species differentiation; these strains were phenotypically classified as *Sporothrix* spp. Interestingly, phenotypic identification of 10 (20%) isolates did not match to the genotypic results. Eight (16%) strains phenotypically classified as *S. schenckii*, DNA sequencing clustered them amid *S. brasiliensis*. The strain phenotypically classified as *S. mexicana* was genotypically identified as *S. schenckii*, and one *S. brasiliensis* was classified as *S. schenckii* by CAL sequencing.

### Mycological, Clinical and Epidemiological Data

**Figure 1:**
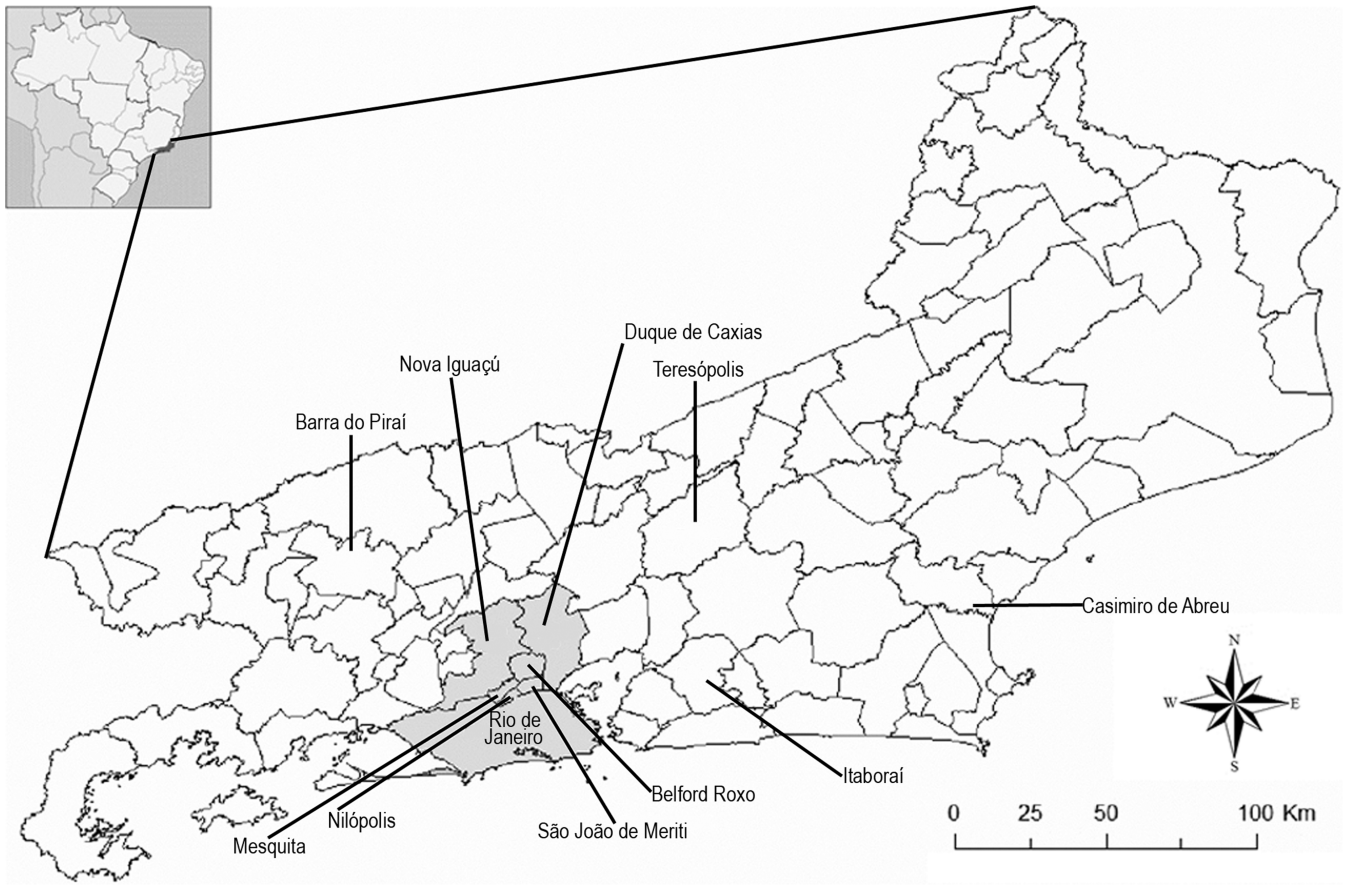
Map of Rio de Janeiro state, Brazil. Names of the cities of origin of the 50 patients included in this study are indicated. Cities in gray, which comprise the Rio de Janeiro metropolitan area, are related to the zoonotic endemic area of sporotrichosis.

Forty-two (93.3%) of the strains identified taxonomically as *S. brasiliensis* were from the Rio de Janeiro endemic area of sporotrichosis, including Rio de Janeiro city, Duque de Caxias, Belford Roxo, Sao João de Meriti, Nova Iguaçu, Nilópolis, and Mesquita (Fig. 1). The other three (6.7%) *S. brasiliensis* strains were isolated from patients who lived in Teresópolis, a county 91 km away from Rio de Janeiro city. *S. brasiliensis* was isolated from 32 of 34 women (94%). Forty (88.9%) patients with *S. brasiliensis* had documented contacts with cats. Two additional *S. brasiliensis*-infected patients (4.4%) reported plant and glass trauma preceding the development of sporotrichosis.

With respect to the five patients with *S. schenckii*, four of them (80%) were isolated from patients who lived in three different rural regions and one urban area (in Itaboraí, Barra do Piraí, Casimiro de Abreu, and Teresópolis, respectively; 45, 100, 127, and 91 km away from Rio de Janeiro city), which are outside of the endemic area. *S. schenckii* was also isolated from a patient who lived within the zoonotic endemic sporotrichosis area in Rio de Janeiro. Three patients were male and two female.

Hypersensitivity reactions such as erythema nodosum or erythema multiforme (10 cases), disseminated cutaneous forms (6 cases), and all but one case of lymphocutaneous sporotrichosis were all attributed to infection with *S. brasiliensis*. Localized cutaneous forms were observed in patients infected with either *S. brasiliensis* (n = 12, 26.7%) or *S. schenckii* (n = 3, 60%). Disseminated disease occurred due to *S. schenckii* in one patient with AIDS, *S. brasiliensis* in two patients with AIDS, and *S. brasiliensis* in one patient without any history of immunosuppression. Finally, there was one case of fixed cutaneous sporotrichosis caused by *S. brasiliensis* in a HIV infected patient with CD4>200 cells/µL.

### Response to Therapy

Four patients infected with *S. brasiliensis* were lost to follow-up. The three patients with AIDS and disseminated disease were excluded from analysis since they received amphotericin B as part of their antifungal regimen. Spontaneous regression was observed in one patient infected with *S. schenckii* (fixed form) and three with *S. brasiliensis* (two fixed and one disseminated cutaneous forms). The remaining 3 cases of *S. schenckii* required more than 24 weeks of itraconazole, and two of them required increased doses (200 and 400 mg/day). Most of the 35 patients infected by *S. brasiliensis* included in this analysis (82.9%) resolved with less than 24 weeks of treatment, regardless of their clinical form. For eight *S. brasiliensis*-infected patients, up to 400 mg/day itraconazole were necessary for clinical cure. The median time to cure for patients with hypersensitivity reactions was similar to the patients without these manifestations (16 weeks), and their diseases resolved with 100 mg/day of itraconazole.

## Discussion

The clinical presentations of sporotrichosis caused by *Sporothrix spp* are highly variable and poorly understood. Kong and collaborators [20] have demonstrated that *S. schenckii* genotypes can be correlated with clinical forms of disease, as mice challenged with *S. schenckii* isolates from patients with fixed cutaneous, lymphocutaneous or disseminated sporotrichosis developed more severe disease according to the severity of the manifestations in the originating patient. However, they did not define the relationships between genotype and treatment outcome or other unusual manifestations. In the present work, we show the direct association between unusual clinical presentations of human sporotrichosis with infection by *S. brasiliensis*. Although *S. brasiliensis* caused typical manifestations of fixed cutaneous and lymphocutaneous sporotrichosis, all 10 patients with hypersensitivity reactions and 6/7 patients with disseminated disease were infected with *S. brasiliensis*. To the best of our knowledge, this is the first work that demonstrates an association between genotypic identification of *Sporothrix* species and several clinical aspects of human sporotrichosis. Given the recent changes in the nomenclature and advances in the molecular taxonomy of *Sporothrix*, it is even more important to understand the clinical implications of these advances [21].

As expected, the majority of our isolates have been identified as *S. brasiliensis* by DNA analyses. Our group has previously characterized *S. brasiliensis* in 230 (93.5%) of 246 isolates obtained from this endemic zoonotic transmission area [8]. A study by Marimon and coworkers of 127 *Sporothrix* strains collected from several parts of the world reported only *S. brasiliensis* among the tested isolates from Rio de Janeiro [4]. There are also a few reports of *S. brasiliensis* in Brazilian states other than Rio de Janeiro [10], [22], [23], but, in these states, the frequency of *S. brasiliensis* appears to be lower than that for the other *Sporothrix* species, with *S. schenckii* predominating [22].

As noted above, *S. brasiliensis* genotype caused typical clinical forms of sporotrichosis (lymphocutaneous and fixed cutaneous). However, except for 1 case of disseminated *S. schenckii* in a patient with AIDS, all of the unusual clinical forms of sporotrichosis were attributed to infection with *S. brasiliensis*, including disseminated cutaneous sporotrichosis in the absence of an underlying immunosuppressive condition, mucosal involvement affecting nasal cavity or conjunctiva, and hypersensitivity reactions. Regarding the hypersensitivity manifestations, our finding are consistent with the previously reported cases of erythema nodosum and erythema multiforme associated with zoonotic sporotrichosis [24], [25] due to *S. brasiliensis*. Recently, Sweet syndrome has also described in 3 patients with sporotrichosis [26], and studies are underway to determine, by calmodulin sequencing, the species involved in these cases.

Another interesting finding for disease due to *S. brasiliensis* is the 32/13 female/male ratio, since there is a predominance of male over female patients with sporotrichosis caused by *S. schenckii*. This can be explained by the fact that the most affected group in the endemic area of sporotrichosis in Rio de Janeiro are housewives that interact with or take care of *S. brasiliensis* infected cats [1].

Barros and coworkers [27] studied the effects of itraconaozle treatment on cutaneous sporotrichosis in 645 patients from the Rio de Janeiro epidemic, including 87 patients with erythema nodosum or erythema multiforme. Interestingly, they observed that of the patients with hypersensitivity reactions resolved their disease more rapidly compared with patients without these conditions. Although our current study did not find differences regarding treatment between these groups, we did determine that most patients with hypersensitivity reactions presented with fixed cutaneous sporotrichosis. Hence, we believe that hypersensitivity reactions may indicate a robust host response to the *S. brasiliensis* yeast cells and play a protective role in sporotrichosis, as observed in coccidioidomycosis [28].

The small number of *S. schenckii* cases in our study calls our attention to the infection caused by this species in Rio de Janeiro. The majority of these cases occurred in rural counties where inhabitants are engaged in agricultural activities, and, therefore, they have frequent and protracted contact with soil. Moreover, in two of these cases, patients denied cat contact. However, *S. schenckii* was identified in one case from the endemic zoonotic transmission area. Our results suggest that *S. schenckii* also circulates, in minor proportions, in this endemic area. New studies with a large number of *S. schenckii* infected patients are necessary to verify the clinical meaningful of sporotrichosis caused by this species.

Several factors could influence the different outcomes of sporotrichosis, such as the size of initial inoculum, the host immune response status, depth of traumatic inoculation and fungal virulence [29]. Virulence studies in a mouse infection model have shown that *S. brasiliensis* is significantly more lethal and results in higher fungal burdens compared to *S. schenckii* as well as other examined *Sporothrix* spp. [30]. This same study concludes that lesional mechanisms could be species-specific, which supports our results. Zoonotic transmission of sporotrichosis by cats results in high *Sporothrix* inoculums for humans, since these animals have high fungal burdens [31]. In some of the endemic sporotrichosis cases, fungal inoculation is presumably repetitive, due to constant bites and scratches suffered by owners [32]. These factors, coupled with the purported higher virulence of *S. brasiliensis* [30], could account for the unusual and more severe clinical manifestations observed with this species.

Itraconazole is the drug of choice for sporotrichosis treatment [27]. It is interesting to note that, regardless the clinical form, there was a trend toward shorter treatment durations in patients with sporotrichosis caused by *S. brasiliensis*, (median = 16 weeks) than the cases due to *S. schenckii* (median = 24 weeks), for our study. Although we are comparing 46 cases of *S. brasiliensis* to only 4 infections due to *S. schenckii*, we propose that our finding might have a therapeutic implication. The response of *S. brasiliensis* to treatment is consistent with the fact that *S. brasiliensis* is more susceptible to antifungal drugs, such as itraconazole, posaconazole, and ravuconazole, than *S. schenckii* [33]. Moreover, previous results of our group, which included eight *S. brasiliensis* from this study, showed that strains from the zoonotic endemic area were highly susceptible to itraconazole [14]. Nevertheless, clinical, randomized studies should be performed to confirm these findings.

Since different *Sporothrix* species appear to be related to distinct clinical manifestations and treatment responses, we propose that speciation should become standard laboratory practice. However, it will require a significant effort to make this recommendation a reality in most clinical laboratories. Phenotypic fungal identification is easier than molecular methods to routinely apply. However, in the present work as well as in a previous study from our team [8] and studies from other groups [10], phenotypic description too often fails to be corroborated by genotypic results. Since the differences between the species of the *S. schenckii* complex were observed at the molecular level [18], we considered DNA sequencing as the gold standard on species identification for the present study. Unfortunately, at present, DNA sequencing is not a suitable methodology for routine clinical laboratories. We recently described a simple and reliable T3B DNA fingerprinting methodology to identify the *S. schenckii* species complex at the DNA level [34], making it an alternative identification methodology for clinical microbiology laboratories.

Our study intended to perform a molecular analysis of the most peculiar clinical cases that we observed in this epidemic as well as typical cases, and also patients presenting from areas outside the sporotrichosis belt of zoonotic transmission, which corresponds to almost 20% of our stored samples. We have checked the medical data from the other 196 patients and they were very similar to the cases we included here. The small size of genotyped strains is a weakness of this study, but in our opinion, these cases illustrate the association of *S. brasiliensis* to the unusual presentations of sporotrichosis. Also, molecular genotyping with sequencing of the calmodulin gene is laborious and expensive (at present), with significant financial impact in our severely limited funding situation.

In conclusion, we have used molecular analysis to clearly demonstrate that *S. brasiliensis* is the primary cause of endemic sporotrichosis in Rio de Janeiro state. Moreover, *S. brasiliensis* causes both classic and unusual manifestations of sporotrichosis, including severe disease in otherwise immunocompetent individuals. We also have documented that local and invasive *S. brasiliensis* disease responds well to itraconazole therapy, with shorter durations of therapy compared to the patients studied with sporotrichosis caused by *S. schenckii*. This study adds new information to our knowledge base on *S. brasiliensis* disease and supports the careful speciation of *Sporothrix* isolates to guide clinical care.

## Supporting Information

Checklist S1 STROBE checklist.
(DOCX)

## References

[1] Barros MBL, Almeida-Paes R, Schubach AO (2011) *Sporothrix schenckii* and Sporotrichosis. Clin Microbiol Rev 24: 633–654. 21976602 10.1128/CMR.00007-11 PMC3194828

[2] Conti Diaz IA (1989) Epidemiology of sporotrichosis in Latin America. Mycopathologia 108: 113–116. 2687693 10.1007/BF00436061

[3] Queiroz-Telles F, Nucci M, Colombo AL, Tobon A, Restrepo A (2011) Mycoses of implantation in Latin America: an overview of epidemiology, clinical manifestations, diagnosis and treatment. Med Mycol 49: 225–236. 21128710 10.3109/13693786.2010.539631

[4] Marimon R, Cano J, Gene J, Sutton DA, Kawasaki M, et al (2007) *Sporothrix brasiliensis*, *S. globosa*, and *S. mexicana*, three new *Sporothrix* species of clinical interest. J Clin Microbiol 45: 3198–3206. 17687013 10.1128/JCM.00808-07 PMC2045377

[5] Cruz R, Vieille P, Oschilewski D (2012) Aislamiento ambiental de *Sporothrix globosa* en relación a un caso de esporotricosis linfo-cutánea. Rev Chilena Infectol 29: 401–405. 23096539 10.4067/S0716-10182012000400006

[6] Madrid H, Cano J, Gene J, Bonifaz A, Toriello C, et al (2009) *Sporothrix globosa*, a pathogenic fungus with widespread geographical distribution. Rev Iberoam Micol 26: 218–222. 19635441 10.1016/j.riam.2009.02.005

[7] Oliveira MME, Almeida-Paes R, Muniz MM, Barros MBL, Gutierrez-Galhardo MC, et al (2010) Sporotrichosis caused by *Sporothrix globosa* in Rio de Janeiro, Brazil: case report. Mycopathologia 169: 359–363. 20131099 10.1007/s11046-010-9276-7

[8] Oliveira MME, Almeida-Paes R, Muniz MM, Gutierrez-Galhardo MC, Zancope-Oliveira RM (2011) Phenotypic and molecular identification of *Sporothrix* isolates from an epidemic area of sporotrichosis in Brazil. Mycopathologia 172: 257–267. 21701792 10.1007/s11046-011-9437-3

[9] Dias NM, Oliveira MME, Santos C, Zancope-Oliveira RM, Lima N (2011) Sporotrichosis caused by *Sporothrix mexicana*, Portugal. Emerg Infect Dis 17: 1975–1976. 22000393 10.3201/eid1710.110737 PMC3310684

[10] Rodrigues AM, de Hoog S, Camargo ZP (2013) Emergence of pathogenicity in the *Sporothrix schenckii* complex. Med Mycol 51: 405–412. 22989196 10.3109/13693786.2012.719648

[11] Marimon R, Gene J, Cano J, Guarro J (2008) *Sporothrix luriei*: a rare fungus from clinical origin. Med Mycol 46: 621–625. 19180753 10.1080/13693780801992837

[12] Freitas DFS, Valle ACF, Almeida Paes R, Bastos FI, Gutierrez-Galhardo MC (2010) Zoonotic Sporotrichosis in Rio de Janeiro, Brazil: a protracted epidemic yet to be curbed. Clin Infect Dis 50: 453. 10.1086/649891 20064034

[13] Silva MB, Costa MM, Torres CC, Gutierrez-Galhardo MC, Valle ACF, et al (2012) Esporotricose urbana: epidemia negligenciada no Rio de Janeiro, Brasil. Cad Saude Publica 28: 1867–1880. 23090167 10.1590/s0102-311x2012001000006

[14] Gutierrez-Galhardo MC, Zancopé-Oliveira RM, Valle ACF, Almeida-Paes R, Tavares PMS, et al (2008) Molecular epidemiology and antifungal susceptibility patterns of *Sporothrix schenckii* isolates from a cat-transmitted epidemic of sporotrichosis in Rio de Janeiro, Brazil. Med Mycol 46: 141–151. 18324493 10.1080/13693780701742399

[15] Reis RS, Almeida-Paes R, Muniz MM, Tavares PMS, Monteiro PCF, et al (2009) Molecular characterisation of *Sporothrix schenckii* isolates from humans and cats involved in the sporotrichosis epidemic in Rio de Janeiro, Brazil. Mem Inst Oswaldo Cruz 104: 769–774. 19820840 10.1590/s0074-02762009000500018

[16] Barros MBL, Schubach AO, Schubach TMP, Wanke B, Lambert-Passos SR (2008) An epidemic of sporotrichosis in Rio de Janeiro, Brazil: epidemiological aspects of a series of cases. Epidemiol Infect 136: 1192–1196. 18028580 10.1017/S0950268807009727 PMC2870916

[17] Barros MBL, Schubach TMP, Gutierrez-Galhardo MC, Schubach AO, Monteiro PCF, et al (2001) Sporotrichosis: an emergent zoonosis in Rio de Janeiro. Mem Inst Oswaldo Cruz 96: 777–779. 11562701 10.1590/s0074-02762001000600006

[18] Marimon R, Gene J, Cano J, Trilles L, Lazera MS, et al (2006) Molecular phylogeny of *Sporothrix schenckii* . J Clin Microbiol 44: 3251–3256. 16954256 10.1128/JCM.00081-06 PMC1594699

[19] Otto TD, Vasconcellos EA, Gomes LH, Moreira AS, Degrave WM, et al (2008) ChromaPipe: a pipeline for analysis, quality control and management for a DNA sequencing facility. Genet Mol Res 7: 861–871. 18949705 10.4238/vol7-3x-meeting04

[20] Kong X, Xiao T, Lin J, Wang Y, Chen HD (2006) Relationships among genotypes, virulence and clinical forms of *Sporothrix schenckii* infection. Clin Microbiol Infect 12: 1077–1081. 17002606 10.1111/j.1469-0691.2006.01519.x

[21] de Hoog GS, Haase G, Chaturvedi V, Walsh TJ, Meyer W, et al (2013) Taxonomy of medically important fungi in the molecular era. Lancet Infect Dis 13: 385–386. 23618329 10.1016/S1473-3099(13)70058-6

[22] Oliveira DC, Lopes PG, Spader TB, Mahl CD, Tronco-Alves GR, et al (2011) Antifungal susceptibilities of *Sporothrix albicans*, *S. brasiliensis*, and *S. luriei* of the *S. schenckii* complex identified in Brazil. J Clin Microbiol 49: 3047–3049. 21653757 10.1128/JCM.00255-11 PMC3147739

[23] Silva-Vergara ML, Camargo ZP, Silva PF, Abdalla MR, Sgarbieri RN, et al (2012) Disseminated *Sporothrix brasiliensis* infection with endocardial and ocular involvement in an HIV-infected patient. Am J Trop Med Hyg 86: 477–480. 22403321 10.4269/ajtmh.2012.11-0441 PMC3284366

[24] Gutierrez-Galhardo MC, Barros MBL, Schubach AO, Cuzzi T, Schubach TMP, et al (2005) Erythema multiforme associated with sporotrichosis. J Eur Acad Dermatol Venereol 19: 507–509. 10.1111/j.1468-3083.2005.01148.x 15987308

[25] Gutierrez-Galhardo MC, Schubach AO, Barros MBL, Moita-Blanco TC, Cuzzi-Maya T, et al (2002) Erythema nodosum associated with sporotrichosis. Int J Dermatol 41: 114–116. 11982651 10.1046/j.1365-4362.2002.01381_2.x

[26] Freitas DFS, Valle ACF, Cuzzi T, Brandao LG, Zancope-Oliveira RM, et al (2012) Sweet syndrome associated with sporotrichosis. Br J Dermatol 166: 212–213. 21711340 10.1111/j.1365-2133.2011.10496.x

[27] Barros MBL, Schubach AO, Oliveira RVC, Martins EB, Teixeira JL, et al (2011) Treatment of cutaneous sporotrichosis with itraconazole - study of 645 patients. Clin Infect Dis 52: e200–206. 21628477 10.1093/cid/cir245

[28] Arsura EL, Kilgore WB, Ratnayake SN (1998) Erythema nodosum in pregnant patients with coccidioidomycosis. Clin Infect Dis 27: 1201–1203. 9827269 10.1086/514985

[29] Carlos IZ, Sassa MF, Sgarbi DBG, Placeres MC, Maia DC (2009) Current research on the immune response to experimental sporotrichosis. Mycopathologia 168: 1–10. 19241140 10.1007/s11046-009-9190-z

[30] Arrillaga-Moncrieff I, Capilla J, Mayayo E, Marimon R, Marine M, et al (2009) Different virulence levels of the species of *Sporothrix* in a murine model. Clin Microbiol Infect 15: 651–655. 19624508 10.1111/j.1469-0691.2009.02824.x

[31] Schubach TMP, Schubach AO, Okamoto T, Barros MBL, Figueiredo FB, et al (2004) Evaluation of an epidemic of sporotrichosis in cats: 347 cases (1998–2001). J Am Vet Med Assoc 224: 1623–1629. 15154732 10.2460/javma.2004.224.1623

[32] Schubach AO, Schubach TMP, Barros MBL (2005) Epidemic cat-transmitted sporotrichosis. N Engl J Med 353: 1185–1186. 10.1056/NEJMc051680 16162897

[33] Marimon R, Serena C, Gene J, Cano J, Guarro J (2008) In vitro antifungal susceptibilities of five species of *Sporothrix* . Antimicrob Agents Chemother 52: 732–734. 18039919 10.1128/AAC.01012-07 PMC2224726

[34] Oliveira MME, Sampaio P, Almeida-Paes R, Pais C, Gutierrez-Galhardo MC, et al (2012) Rapid identification of *Sporothrix* species by T3B fingerprinting. J Clin Microbiol 50: 2159–2162. 22403427 10.1128/JCM.00450-12 PMC3372112

